# Apoptotic vesicles rescue impaired mesenchymal stem cells and their therapeutic capacity for osteoporosis by restoring miR-145a-5p deficiency

**DOI:** 10.21203/rs.3.rs-4416138/v1

**Published:** 2024-06-04

**Authors:** Rong Zhang, Xiaodan Mu, Dawei Liu, Chider Chen, Bowen Meng, Yan Qu, Jin Liu, Runci Wang, Chuanjie Li, Xueli Mao, Qintao Wang, Qingbin Zhang

**Affiliations:** Guangzhou Medical University; Capital Medical University; Peking University School & Hospital of Stomatology; University of Pennsylvania; Sun Yat-sen University; Sun Yat-sen University; Sichuan University; University of Pennsylvania; Guangzhou Medical University; Sun Yat-sen University; Air Force Medical University; Guangzhou Medical University

**Keywords:** Apoptotic vesicles, Mesenchymal stem cells, Osteoporosis, miR-145a-5p, TGF-β/Smad signaling, Wnt/β-catenin signaling

## Abstract

Apoptotic vesicles (apoVs) play a vital role in various pathological conditions; however, we have yet to fully understand their precise biological effects in rescuing impaired mesenchymal stem cells (MSCs) and regulating tissue homeostasis. Here, we proved that systemic infusion of bone marrow MSCs derived from wild-type (WT) mice effectively improved the osteopenia phenotype and hyperimmune state in ovariectomized (OVX) mice. Importantly, the WT MSCs rescued the impairment of OVX MSCs both *in vivo* and *in vitro*, whereas OVX MSCs did not show the same efficacy. Interestingly, treatment with apoVs derived from WT MSCs (WT apoVs) restored the impaired biological function of OVX MSCs and their ability to improve osteoporosis. This effect was not observed with OVX MSCs-derived apoVs (OVX apoVs) treatment. Mechanistically, the reduced miR-145a-5p expression hindered the osteogenic differentiation and immunomodulatory capacity of OVX MSCs by affecting the TGF-β/Smad 2/3-Wnt/β-catenin signaling axis, resulting in the development of osteoporosis. WT apoVs directly transferred miR-145a-5p to OVX MSCs, which were then reused to restore their impaired biological functions. Conversely, treatment with OVX apoVs did not produce significant effects due to their limited expression of miR-145a-5p. Overall, our findings unveil the remarkable potential of apoVs in rescuing the biological function and therapeutic capability of MSCs derived from individuals with diseases. This discovery offers a new avenue for exploring apoVs-based MSC engineering and expands the application scope of stem cell therapy, contributing to the maintenance of bone homeostasis through a previously unrecognized mechanism.

## Introduction

Osteoporosis is a chronic metabolic bone disease caused by various reasons, characterized by decreased bone density and quality, damaged bone microstructure, and increased bone fragility, which adversely affect the patients’ life quality and bring a huge burden to individuals, families, and society. Current therapeutic approaches for osteoporosis encompass the use of vitamin D and calcium agents, nonsteroidal antiinflammatory drugs (NSAIDs), selective estrogen receptor modulator (SERM), estrogen replacement therapy (ERT), bisphosphonate therapy, and parathyroid hormone therapy [1-4]. While these treatments can provide some relief from clinical symptoms, they inevitably lead to significant side effects, including an increased risk of cancer, myocardial infarction, thromboembolism, and osteonecrosis of the jaw [5, 6].

In recent years, stem cell therapy has received widespread attention owing to its remarkable efficacy in treating numerous diseases [7, 8]. For instance, it has shown effectiveness in ameliorating bone loss in various animal models with minimal side effects [9, 10]. The underlying mechanisms involve the paracrine action of cytokines, cell-to-cell contact, and the release of extracellular vesicles, which is currently a major research focus [11-13]. In our early studies, we utilized human bone marrow mesenchymal stem cells (BMMSCs) and exfoliated deciduous tooth stem cells (SHED) in the treatment of an osteoporosis mouse model induced by ovariectomy (OVX). We demonstrated that the therapeutic mechanism relied on the Fas/FasL-mediated killing of MSCs on recipient T cells and the inhibition of osteoclast differentiation [9, 14]. Furthermore, other researchers have also reported the beneficial therapeutic effects of MSCs on osteoporosis [10, 15-17]. However, our previous experiments revealed no significant effect when using MSCs derived from OVX mice for the treatment of osteoporosis (unpublished data), leaving the specific mechanism unclear. This limitation restricts the potential cell sources for stem cell therapy and hampers the advancement of relative techniques. In view of this, various measures have been taken to enhance MSCs' functionality, including genetic engineering, or preconditioning with specific drugs or biological factors [18, 19]. Nonetheless, concerns regarding biological safety, duration of effects, and ethical considerations necessitate the development of novel approaches for MSC engineering.

Cells in the human body undergo apoptosis at all times, which is an essential metabolic process crucial for maintaining tissue and organ homeostasis [20-22]. This process generates a substantial number of apoptotic vesicles (apoVs) [23-25], which possess distinct molecular characteristics compared to exosomes and microvesicles [26-29]. ApoVs mediate intercellular communication by releasing and delivering various components, including DNAs, RNAs, lipids, and organelles inherited from parent cells. These components can exert therapeutic effects either by direct contact with living cells or through endocytosis by recipient cells [30, 31]. Notably, emerging evidence suggests that transplanted MSCs will soon undergo apoptosis, and the resulting apoptotic vesicles are the key to exerting therapeutic effects [28, 32]. Previous research from our group has provided substantial evidence supporting the unique therapeutic effects of systemically infused apoVs derived from exogenous MSCs in various diseases, including haemophilia, osteoporosis, arthritis, skin injuries, hair regeneration, and tumors [22, 33-36]. Importantly, MSCs-derived apoVs possess advantages such as low immunogenicity, long blood circulation time, easy storage, abundant production, and potential for drug delivery vehicles engineering [37, 38]. Furthermore, our previous findings demonstrate that apoVs can inherit pluripotent molecules from embryonic stem cells (ESCs) to rejuvenate adult stem cells, suggesting their potential for modifying MSCs [39]. However, the specific effects of apoVs on impaired MSCs or MSCs derived from individuals with diseases, as well as the underlying mechanisms, still need to be elucidated.

In this research, we proved apoVs from healthy individuals possessed the ability to rescue the impaired biological function of MSCs and enhance their effectiveness in improving osteoporosis. Furthermore, we conducted a detailed investigation into the underlying mechanism and found that it may involve the cargo miR-145a-5p and its subsequent reuse to restore its deficiency.

## Results

### Characterization of MSCs-derived apoVs

Initially, we employed conventional methods to isolate, culture, and characterize mouse BMMSCs. The cells displayed the characteristic morphology (Fig. S1A) and demonstrated colony-forming ability, as well as the capacity for osteogenic and adipogenic differentiation (Fig. S1B-D). Additionally, the cells exhibited positive expression of markers associated with mouse MSCs (CD73, CD90, CD105, CD126, CD166, and Scal-1) and negative expression of markers related to hematopoietic stem cells (CD34, CD45) (Fig. S1E).

**Figure 1:**
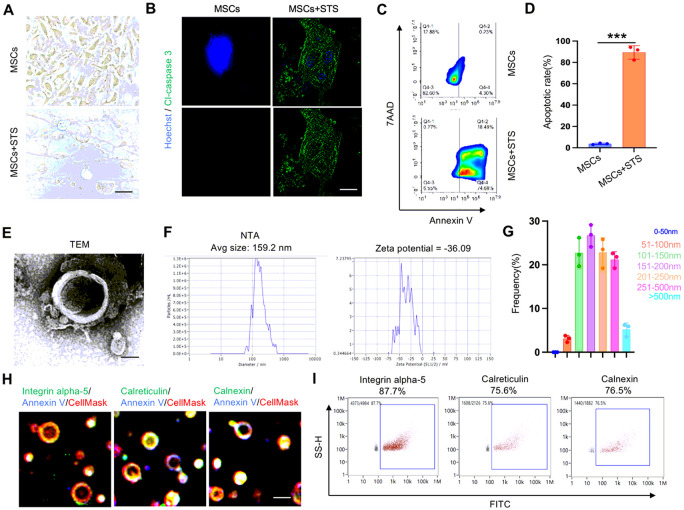
Characterization of apoptotic vesicles (apoVs) from MSCs. (**A**) The morphological change of MSCs after 16h STS treatment as observed by microscopy. **(B)** Immunofluorescent staining displayed the apoptotic cells in which cleaved caspase 3 was stained by green fluorescence after STS treatment. (**C, D**) Flow cytometry analysis showed the apoptotic cells after STS induction as indicated by the proportion of Annexin V^+^ 7AAD^+^ cells. (**E**) Representative transmission electron microscopy (TEM) image demonstrated the typical morphology of apoVs. (**F, G**) NTA assay showed the size distribution and zeta potential of apoVs. (**H, I**) Immunofluorescent staining and nanoflow cytometry showed the positive expression of apoVs specific markers integrin alpha-5, calreticulin and calnexin together with the apoptotic marker annexin V on apoVs. n = 3. *** *P* < 0.001. Data are presented as mean ± SD. Scale bar, 100 μm (**A**), 50 μm (**B**), 25 nm (**E**), 100 m (**H**).

Subsequently, we isolated and characterized apoVs derived from mouse MSCs following the methodology outlined in our previous study [36]. To initiate apoptosis in MSCs, we utilized staurosporine (STS) and isolated apoVs through a carefully optimized gradient centrifugation procedure (Fig. S2). The cells displayed typical morphological changes and apoptotic responses after 16 hours of STS treatment, as observed through microscopy, immunofluorescent staining of cleaved-caspase 3, and flow cytometry analysis of annexin V and 7AAD expression (Fig. 1A-D). Representative transmission electron microscopy (TEM) images provided visual confirmation of the characteristic morphology of apoVs (Fig. 1E). To validate the size distribution of apoVs, we conducted a nanoparticle track analysis (NTA) assay (Fig. 1F and G). Moreover, nano flow cytometry and immunofluorescent staining analysis revealed significant expression of integrin alpha-5 (87.7%), calreticulin (75.6%), and calnexin (76.5%) in apoVs (Fig. 1H and I).

#### WT apoVs rather than OVX apoVs rescued the impaired therapeutic effect of OVX MSCs on osteoporosis

**Figure 2:**
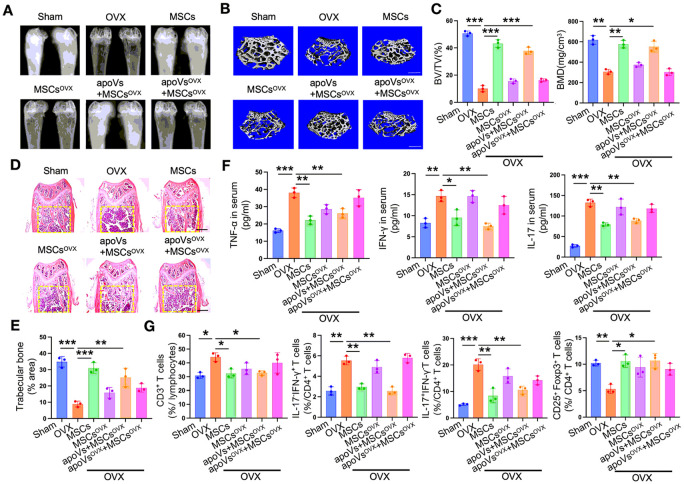
WT apoVs effectively rescued the impaired therapeutic effect of OVX MSCs on osteoporosis as depicted by the recovery of osteoporotic phenotype and hyperimmune state in OVX mice, rather than OVX apoVs. (**A**) X-ray image showed that the bone density of femurs was significantly reduced in OVX mice, which was restored after 4 weeks of WT MSCs infusion. WT apoVs rather than OVX apoVs treatment obviously rescued the restorative effect of OVX MSCs. (**B, C**) Micro CT analysis showed a similar phenomenon as the X-ray image depicted, indicated by bone volume/total volume (BV/TV) and bone mineral density (BMD). (**D, E**) H&E staining showed a similar volume of trabecular bone in the OVX MSCs+WT apoVs group as compared with the WT MSCs group, which could not be seen in the OVX MSCs group and OVX MSCs+OVX apoVs group. (**F**) ELISA assay proved that WT apoVs treatment recovered the capacity of OVX MSCs to ameliorate the over-expressed Th1 and Th17 subsets related to proinflammatory factors TNF-α, IFN-γ, and IL-17 in the serum of OVX mice. (**G**) Flow cytometry analysis demonstrated that the proportion of CD3^+^ T cells in splenic lymphocytes, the Th1/Th17 subsets in CD4^+^ T cells of splenocytes were all reduced, while the Treg subsets were elevated in the WT MSCs and OVX MSCs+WT apoVs group. n = 3. * *P* < 0.05, ** *P* < 0.01, *** *P* < 0.001. Data are presented as mean ± SD. Scale bar, 500 μm (**B**), 1 mm (**D**).

Analysis of X-ray and micro-CT scans revealed a significant decrease in local bone density and cancellous bone mass in the distal femurs of the OVX group compared to the sham group (Fig. 2A and B), indicating successful modeling. Following 4 weeks of WT MSCs treatment, there was a remarkable recovery in bone density, cancellous bone mass, bone volume/total volume (BV/TV), and bone mineral density (BMD) like that of the sham group. However, the BMD only exhibited a slight rebound in the OVX MSCs group, which was significantly lower than that of the WT MSCs group (Fig. 2A-C). These findings suggest that WT MSCs treatment effectively alleviates osteopenia symptoms in OVX mice, whereas treatment with OVX MSCs is ineffective. Importantly, it should be noted that when OVX MSCs were preconditioned *in vitro* with WT apoVs for 3 days and then systemically infused, there was a substantial increase in bone density, trabecular bone mass, BMD, and BV/TV comparable to the WT MSCs group. In contrast, the OVX apoVs group failed to show a similar effect (Fig. 2A-C). This indicates that *in vitro* stimulation with WT apoVs restores the impaired capability of OVX MSCs to effectively treat osteoporosis, rather than OVX apoVs.

The HE staining of distal femurs in mice revealed distinct findings. In comparison to the sham group, the OVX group exhibited a reduction in trabecular bone density, with most of the remaining trabecular bone being discontinuous. Additionally, there was a prominent presence of immune cell infiltration in the bone marrow cavity. Conversely, the WT MSCs group demonstrated mostly continuous and interwoven trabecular bone, with a significant increase in both total amount and density. Furthermore, only a small amount of infiltrating immune cells w observed. However, these positive effects were not observed in the OVX MSCs group. Interestingly, the beneficial outcomes of OVX MSCs were nearly equivalent to those of the WT MSCs group when subjected to preconditioning with WT apoVs. Conversely, treatment with OVX apoVs did not yield similar results (Fig. 2D and E).

Given that osteoporosis is a chronic inflammatory disease associated with a prolonged hyperimmune state, we proceeded to investigate the changes in CD3^+^ T cells, Treg cells (regulatory T cells), and Th cells (helper T cells) subsets following systemic infusion of MSCs. Through ELISA and flow cytometry analysis, we demonstrated that treatment with WT apoVs restored the ability of OVX MSCs to mitigate the overexpressed proinflammatory factors TNF-α, IFN-γ, and IL-17, associating with Th1 and Th17 cells, in the serum of OVX mice. Additionally, WT apoVs treatment reduced the excessive activation of CD3^+^ T cells and the Th1/Th17 subsets in splenic lymphocytes, while simultaneously elevating the depleted Treg subsets. A similar pattern was observed in the WT MSCs group, but no such effects were evident in the OVX MSCs and OVX MSCs + OVX apoVs group (Fig. 2F and G).

#### WT apoVs rather than OVX apoVs rescued the therapeutic effect of impaired OVX MSCs on the recovery of recipient MSCs in osteoporotic mice

**Figure 3:**
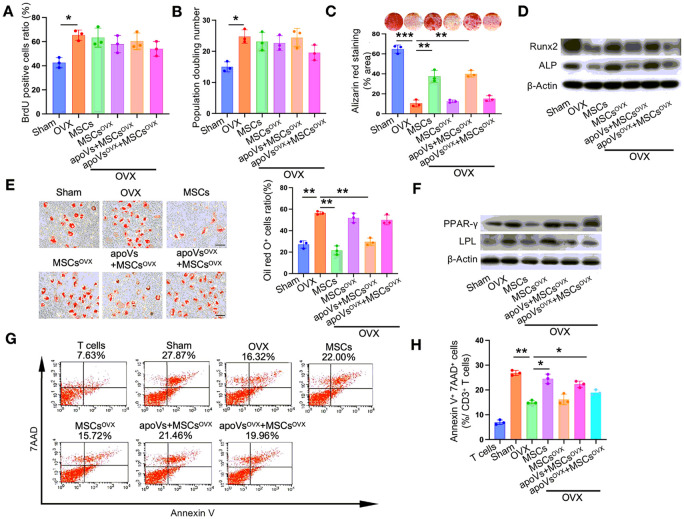
WT apoVs obviously rescued the impaired therapeutic capacity of OVX MSCs on the recovery of osteogenic differentiation, adipogenic differentiation and immunoregulatory properties of MSCs derived from the osteoporotic mice, rather than OVX apoVs. (**A, B**) BrdU labeling and continuous passage assay showed that MSCs from the OVX mice proliferated at a higher rate. Exogenous MSC infusion had no obvious effect on the proliferation of recipient MSCs from the OVX mice. (**C**) Alizarin red staining showed the recovery of osteogenic differentiation capacity of recipient MSCs from the OVX mice after exogenous infusion of WT MSCs and WT apoVs preconditioned OVX MSCs. (**D**) Western blot assay evaluating the expression of osteogenic differentiation-related protein Runx2 and ALP showed similar results as (**C**). (**E**) Oil red O staining manifested the recovery of adipogenic differentiation capacity of recipient MSCs from the OVX mice after exogenous infusion by the WT MSCs and WT apoVs preconditioned OVX MSCs. (**F**) Western blot assay evaluating the expression of adipogenic differentiation-related protein PPAR-γ and LPL showed similar results as (**E**). (**G, H**) Flow cytometric analysis showed that the WT apoVs preconditioned OVX MSCs infusion effectively rescued the immunoregulatory capacity of recipient MSCs, indicating inducing a similar amount of T lymphocytes apoptosis compared to the WT MSCs infusion. n = 3. * *P* < 0.05, ** *P* < 0.01, *** *P* < 0.001. Data are presented as mean ± SD. Scale bar, 50 μm (**E**).

It is widely acknowledged that stem cells play an indispensable role in maintaining tissue homeostasis [40-42]. Therefore, our next objective was to determine whether MSCs treatment could rescue the impaired recipient MSCs in osteoporotic mice. We isolated BMMSCs from the OVX mice who received systemic MSCs infusion and assessed their key biological functions. The BrdU labeling and continuous passage assays revealed an increase in the proliferation and population doubling rate of MSCs derived from the OVX mice. However, the infusion of MSCs failed to change the proliferation and population doubling rate of MSCs originating from the OVX mice (Fig. 3A and B).

Regarding the differentiation property, we demonstrated that WT apoVs effectively restored the therapeutic impact of OVX MSCs on the restoration of osteogenic and adipogenic differentiation capacity to a similar degree as WT MSCs. However, OVX apoVs did not exhibit such an effect. This was evident from the formation of calcium nodules and lipid droplets, as well as the expression of osteogenic markers including runt-related transcription factor 2 (Runx2) and alkaline phosphatase (ALP), and adipogenic markers, peroxisome proliferator-activated receptor y (PPARy) and lipoprotein lipase (LPL) (Fig. 3C-F).

Given the crucial role of their MSCs in regulating the host immune system and maintaining immunological homeostasis [43, 44], we investigated the immunoregulatory capacity of recipient MSCs following the administration of exogenous MSCs. It was observed that preconditioning with WT apoVs, in contrast to OVX apoVs, restored the reparative effect of exogenous OVX MSCs on the recovery of recipient MSCs' immunoregulatory capacity. This was evident in the induction of T cell apoptosis comparable to that showed in the WT MSCs group (Fig. 3G and H).

#### WT apoVs rather than OVX apoVs recovered the impaired biological functions of OVX MSCs in vitro

**Figure 4:**
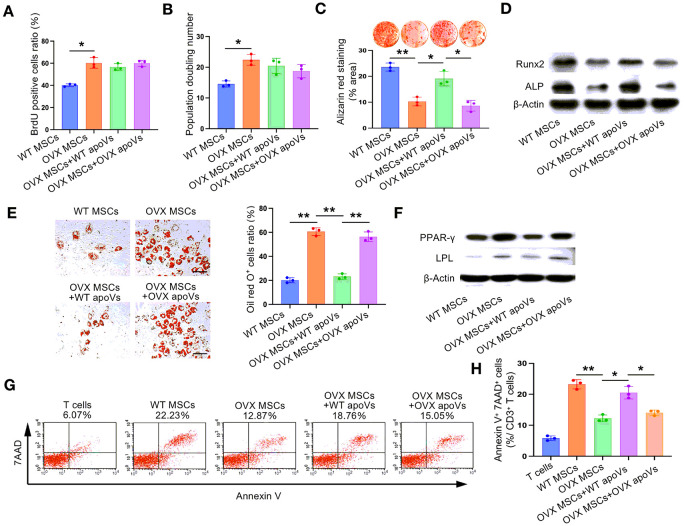
WT apoVs prominently remedy the impaired osteogenic differentiation, adipogenic differentiation and immunoregulatory properties of OVX MSCs in vitro, other than OVX apoVs. **(A, B)** BrdU labeling and continuous passage assay demonstrated that apoVs treatment had no obvious effect on the proliferation of OVX MSCs. **(C)** Alizarin red staining showed the recovery of osteogenic differentiation capacity of OVX MSCs after WT apoVs treatment, other thanOVX apoVs. **(D)** Western blot assay indicated that the expression of osteogenic differentiation-related protein Runx2 and ALP was extremely elevated after WT apoVstreatment, instead of OVX apoVs treatment. **(E)** Oil red O staining manifested the recovery of adipogenic differentiation capacity of OVX MSCs stimulated by WT apoVs, rather than OVX apoVs. **(F)** Western blot assay indicated that the expression of adipogenic differentiation-related protein PPAR-γ and LPL was significantly reduced after WT apoVstreatment, but not OVX apoVs treatment. **(G, H)** Flow cytometric analysis demonstrated that WT apoVs treatment remarkablyimproved the immunoregulation capacity of OVX MSCs, while the OVX apoVs treated group showed no similar effect. n = 3. * *P* < 0.05, ** *P* < 0.01. Data are presented as mean ± SD. Scale bar, 50 μm **(E)**.

Based on the observation that WT apoVs effectively restored the therapeutic effect of OVX MSCs on osteoporosis, we were intrigued to investigate whether apoVs could have a similar effect on damaged cells in vitro. Therefore, we assessed the proliferation, osteogenic and adipogenic differentiation, and immunoregulatory properties of OVX MSCs after 3 days of apoVs treatment. Interestingly, it was found that apoVs did not impact the proliferation and population doubling rate of OVX MSCs (Fig. 4A and B). However, in line with our expectations, WT apoVs, but not OVX apoVs, promoted osteogenic differentiation and inhibited adipogenic differentiation of OVX MSCs. This was evident from the increased mineralization nodules formation, elevated ALP and Runx2 expression, decreased lipid droplet formation, and reduced LPL and PPARy expression (Fig. 4C-F), resembling the characteristics of the WT MSCs group. Furthermore, flow cytometry analysis demonstrated that WT apoVs restored the immunoregulatory capacity of OVX MSCs to a level similar to that of WT MSCs, while OVX apoVs did not produce the same effect (Fig. 4G and H). These results indicated that WT apoVs possessed the ability to transform damaged cells into a relatively normal state similar to WT cells.

#### The crosstalk of TGF-β/Smad 2/3 and Wnt/β-catenin signaling pathway plays a central role in the reparative process of WT apoVs on the OVX MSCs

**Figure 5:**
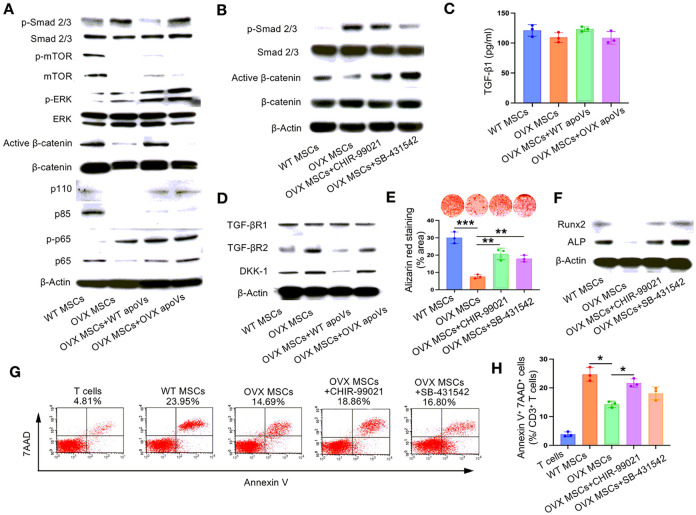
The crosstalk of TGF-β1/Smad 2/3 and Wnt/β-catenin signaling pathway was responsible for the reparative process of WT apoVs on OVX MSCs. (**A**) Western blot assay showed that the TGF-β1/Smad 2/3 signaling pathway was activated while the Wnt/β-catenin signaling pathway was inhibited in OVX MSCs, which was counter-regulated by WT apoVs. (**B**) Western blot assay demonstrated that the Wnt/β-catenin signaling pathway was upregulated after TGF-β1/Smad 2/3 signaling pathway was downregulated in OVX MSCs, which was not feasible conversely. (**C**) ELISA assay showed TGF-β1 secretion remained stable before and after apoVs treatment. (**D**) Western blot assay indicated that WT apoVs treatment obviously decreased the expression of TGF-βR2 and DKK-1. (**E, F**) Alizarin red staining and western blot assay both showed that osteogenic differentiation was promoted after the downregulation of the TGF-β1/Smad 2/3 pathway or the upregulation of the Wnt/β-catenin pathway. (**G, H**) Flow cytometric analysis demonstrated that when the Wnt/β-catenin pathway was activated, the OVX MSCs showed more prominent immunoregulatory capacity. n = 3. * *P* < 0.05, ** *P* < 0.01, *** *P* < 0.001. Data are presented as mean ± SD.

After observing the beneficial reparative effects of WT ApoVs on diseased MSCs in both *in vitro* and *in vivo* settings, further exploration into the underlying mechanisms is warranted. To explore this, we conducted a western blot analysis to assess the activation status of several key signaling pathways associated with osteogenic differentiation and inflammation, namely TGF-β/Smad 2/3, mTOR, ERK, Wnt/β-catenin, PI3K/AKT, and NFkB pathways. Our findings revealed that the TGF-β/Smad 2/3 signaling was activated, while the Wnt/β-catenin signaling was inhibited in OVX MSCs compared to WT MSCs. Interestingly, treatment with WT ApoVs restored the abnormal activation state of the aforementioned signaling pathways, while OVX ApoVs failed to produce a similar effect. Since the changes in other signaling pathways did not align with the observed phenotypic alterations in cells, we deemed the TGF-β/Smad 2/3 and Wnt/β-catenin pathways as the main focus of our study for further investigation (Fig. 5A).

We proceeded to investigate the reciprocal relationship between the two signaling pathways. To achieve this, we utilized CHIR-99021, a GSK-3β inhibitor, to activate Wnt/β-catenin signaling, and SB-431542, a Src family kinase inhibitor, to inhibit TGF-β/Smad 2/3 signaling. Through this experimental approach, we demonstrated that downregulating TGF-β/Smad 2/3 signaling in OVX MSCs subsequently led to upregulation of Wnt/β-catenin signaling. However, the opposite scenario was not feasible. These findings indicated that, in OVX MSCs, TGF-β/Smad 2/3 signaling acted upstream of Wnt/β-catenin signaling (Fig. 5B).

As the TGF-β/Smad 2/3 signaling pathway follows a typical receptor-ligand binding activation mode, our next objective was to investigate the activation process of this pathway in OVX MSCs. Initially, we examined the expression level of TGF-β1 in the supernatant of culture medium, and our findings revealed that apoVs did not have any impact on TGF-β1 secretion in MSCs (Fig. 5C). Therefore, we directed our focus towards the effect of apoVs on the expression of TGF-β receptors and Dickkopf 1 (Dkk1), a significant inhibitor of the Wnt/β-catenin signaling pathway. Our results showed that while the expression of TGF-β receptor 1 (TGF-βR1) remained constant in the four groups, TGF-β receptor 2 (TGF-βR2) was increased in OVX MSCs, but this increase was mitigated by treatment with WT apoVs. Similar findings were observed regarding the expression level of Dkk1 (Fig. 5D). Thus, WT apoVs exhibited inhibitory effects on the TGF-β/Smad 2/3 signaling pathway while simultaneously activating the Wnt/β-catenin signaling pathway through the downregulation of TGF-βR2 and DKK-1.

Subsequently, we proceeded to evaluate the distinct effects of these two signaling pathways on the osteogenic differentiation and immunoregulatory capacity of OVX MSCs. Remarkably, it was observed that either inhibiting the TGF-β/Smad 2/3 signaling pathway or promoting the Wnt/β-catenin signaling pathway effectively facilitated the osteogenic differentiation in OVX MSCs (Fig. 5E and F). Furthermore, the immunoregulatory capacity was restored when the Wnt/β-catenin signaling pathway was promoted (Fig. 5G and H). These findings suggested that the aforementioned signaling pathways may play a crucial role in shaping the phenotype of osteoporosis by regulating the osteogenic differentiation and immunoregulatory capacity of the host MSCs.

### miR-145a-5p was responsible for the WT apoVs-mediated rescue of impaired OVX MSCs in vitro

**Figure 6:**
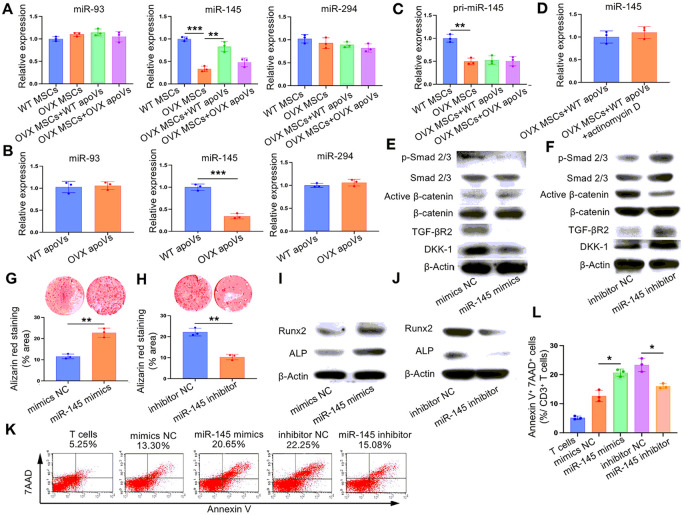
miR-145a-5p contributed to the WT apoVs-mediated rescue of impaired OVX MSCs. (**A**) Real-time PCR analysis indicated that miR-145a-5p expression was decreased in OVX MSCs as compared with WT MSCs, which was increased when treated with WT apoVs *in vitro*. (**B**) Real-time PCR analysis indicated that miR-145a-5p expression was decreased in OVX apoVs compared to WT apoVs. (**C**) Real-time PCR analysis showed that pri-miR-145a-5p expression was downregulated in OVX MSCs compared to WT MSCs, which was unchanged after apoVs stimulation. (**D**) Real-time PCR analysis indicated that actinomycin D treatment failed to affect the miR-145a-5p expression in WT apoVs treated OVX MSCs. (**E, F**) Western blot assay indicated that miR-145a-5p mimics downregulated TGF-β1/Smad2/3 signaling pathway and upregulated Wnt/β-catenin signaling pathway in OVX MSCs, while miR-145a-5p inhibitor displayed the opposite effect in WT MSCs. (**E, F**) Alizarin red staining showed that miR-145a-5p mimics promoted the mineralized nodule formationin OVX MSCs, while miR-145a-5p inhibitor displayed the opposite effect in WT MSCs. (**I, J**) Western blot assay indicated that miR-145a-5p mimics promoted the expression of osteogenic differentiation-related protein Runx2 and ALP in OVX MSCs, while miR-145a-5p inhibitor displayed the opposite effect in WT MSCs. (**K, L**) Flow cytometric analysis indicated that miR-145a-5p mimics upregulated the immunoregulatory capacity of OVX MSCs, while miR-145a-5p inhibitor displayed the opposite effect in WT MSCs. n = 3. * *P* < 0.05, ** *P* < 0.01, *** *P* < 0.001. Data are presented as mean ± SD.

A multitude of studies have provided evidence that apoVs and other extracellular vesicles exert their regulatory functions by transmitting internally encapsulated miRNAs, thus influencing the expression of specific target genes in recipient cells [45-47]. With this understanding, we employed three widely utilized miRNA target gene prediction databases, namely Targetscan, miRDB, and miRWalk, to identify potential miRNAs that target TGF-βR2. Through an intersection of the search results, three miRNAs (miR-93-5p, miR-145a-5p, and miR-294-3p) emerged as promising candidates (Fig. S3). Subsequently, we validated the expression of these three miRNAs in different cell groups and apoVs using real-time PCR. Our findings indicated a significant decrease in miR-145a-5p levels in OVX MSCs and OVX apoVs compared to WT MSCs and WT apoVs, respectively. Conversely, when treated with WT apoVs instead of OVX apoVs, the expression of miR-145a-5p increased, which contrasted with the observed variation in TGF-βR2 expression across the groups. Nevertheless, there is no statistically significant differences in the expression of miR-93-5p and miR-294-3p among the various cell groups and apoVs (Fig. 6A and B).

To confirm the mechanism by which apoVs influence miR-145a-5p expression, we initially evaluated the expression of the primary transcript of miR-145a-5p (pri-miR-145a-5p) in the different cell groups. Our findings demonstrated that OVX MSCs exhibited lower levels of pri-miR-145a-5p, which kept stable following apoVs treatment (Fig. 6C). In addition, we utilized actinomycin D to inhibit RNA synthesis and observed no discernible changes in miR-145a-5p expression (Fig. 6D). the results suggest that the decrease in pri-miR-145a-5p synthesis in OVX MSCs leads to reduced expression of mature miR-145a-5p after cleavage. Importantly, apoVs treatment did not significantly influence pri-miR-145a-5p synthesis, indicating that WT apoVs primarily enhance the expression of mature miR-145a-5p in OVX MSCs through direct cargo delivery and reuse.

Subsequently, we sought to dissect the prospective effect of miR-145a-5p on the TGF-β/Smad 2/3 and Wnt/β-catenin signaling pathways, also on the osteogenic differentiation and immune regulation in OVX MSCs. To assess the functionality of miR-145a-5p, we utilized micro-RNA mimics and inhibitors to manipulate its expression. As demonstrated in Fig. 6E-L, when miR-145a-5p was upregulated using mimics, there was a reduction in the expression of TGF-βR2 and Dkk1. Consequently, the TGF-β/Smad 2/3 signaling pathway was inhibited, while the Wnt/β-catenin signaling pathway was promoted, mediating the restoration of osteogenic differentiation and immune regulation. Conversely, the observed trends were reversed when miR-145a-5p was suppressed using an inhibitor.

#### miR-145a-5p was critical in the apoVs-mediated rescue of the therapeutic effect of OVX MSCs on osteoporosis in vivo

**Figure 7:**
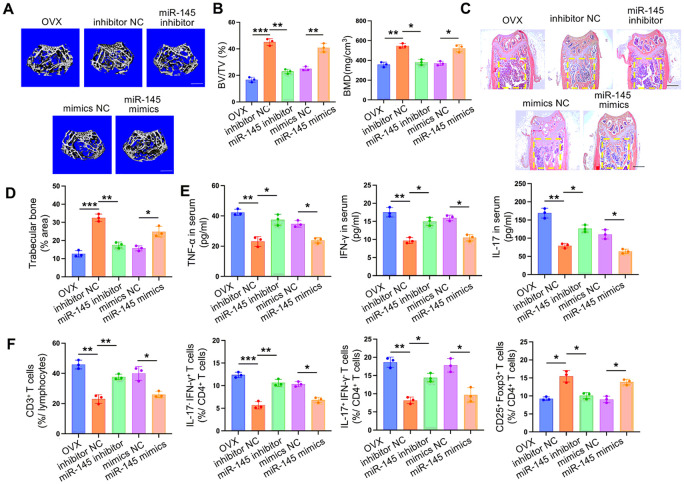
miR-145a-5p is critical in the apoVs-mediated rescue of osteoporotic phenotype and hyperimmune state in OVX mice. (**A, B**) MicroCT analysis showed that when miR-145a-5p expression was inhibited in WT MSCs, its reparative effect on BMD and BV/TV of femurs in OVX mice was significantly abrogated, and the opposite outcome could be seen when miR-145a-5p expression was promoted in OVX MSCs. (**C, D**) H&E staining showed that trabecular bone volume was downregulated in the miR-145a-5p inhibitor preconditioned WT MSCs group as compared with the inhibitor NC preconditioned WT MSCs group, while upregulated in the miR-145 mimics preconditioned OVX MSCs group as compared with the mimics NC preconditioned OVX MSCs group. (**E**) ELISA assay proved that miR-145 inhibitor treatment abolished the effect of WT MSCs to ameliorate the over-expressed Th1 and Th17-related proinflammatory factors TNF-α, IFN-γ, and IL-17 in OVX mice, and the opposite outcome could be seen when the miR-145a-5p expression was promoted in OVX MSCs. (**F**) Flow cytometry analysis demonstrated that the proportion of CD3^+^ T cells in splenic lymphocytes, the Th1/Th17 subsets in CD4^+^ T cells of splenocytes were all elevated while the Treg subsets were reduced when miR-145a-5p expression was inhibited in WT MSCs, and the opposite outcome could be seen when miR-145a-5p expression was promoted in OVX MSCs. n = 3. * *P* < 0.05, ** *P* < 0.01, *** *P* < 0.001. Data are presented as mean ± SD. Scale bar, 200 μm (**A**), 1 mm (**C**).

Based on our data, which demonstrated the involvement of miR-145a-5p in the restoration of impaired OVX MSCs mediated by WT apoVs in vitro, we formulated the hypothesis that the apoVs-mediated rescue of the therapeutic effects of OVX MSCs on osteoporosis might be partially dependent on miR-145a-5p. To investigate this, we manipulated the expression of miR-145a-5p using mimics and an inhibitor, revealing that the overexpressed miR-145a-5p in OVX MSCs significantly enhanced their therapeutic effect on the osteopenic phenotype. This was evident from microCT scans (Fig. 7A and B) and H&E staining (Fig. 7C and D) of distal femurs in OVX mice, as well as from ELISA (Fig. 7E) and flow cytometry analysis (Fig. 7F) indicating improvements in the hyperimmune state of the host. Conversely, the inhibition of miR-145a-5p in WT MSCs yielded opposite results, as the beneficial effects associated with the infusion of WT MSCs were significantly diminished, reaching levels comparable to OVX MSCs treatment (Fig. 7A-F). All evaluation indicators were consistent with those presented in Fig. 2. These findings strongly suggested that miR-145a-5p is indispensable in the apoVs-mediated rescue of the therapeutic effects of OVX MSCs in the context of osteoporosis.

## Discussion

Osteoporosis, the most prevalent chronic metabolic bone disease, poses a significant and urgent problem due to its high risk of fragility fractures. Transplantation of MSCs is considered an ideal treatment method for osteoporosis. This approach holds the promise of increasing the differentiation of osteoblasts while blocking the activation of osteoclasts, thereby rebalancing the processes of bone formation and resorption [48-50]. To enhance the therapeutic effect and efficacy of MSC transplantation, various techniques, such as targeted modification, preconditioning, and co-transplantation, have been employed [10, 51, 52]. Prolonged disease duration can lead to dysfunction of MSCs, disrupting tissue homeostasis [53, 54]. In our previous unpublished study, we discovered that MSCs from individuals with osteoporosis exhibited impaired biological functions and therapeutic effects on osteoporosis. It has been reported that exosomes have the potential to modify MSC functions *in vitro* [55]. However, the application of exosomes derived from normal living cells is limited due to constraints in physiological secretion. Alternatively, ApoVs present a promising new method for *in vitro* modification of stem cells. ApoVs have abundant sources, yield large quantities, undergo concise extraction procedures, and are easily subject to quality control. Therefore, this study delves into the detailed biological role of ApoVs in rescuing the function of MSCs from OVX individuals, aiming to maintain bone homeostasis through the delivery of ApoV-encapsulated miR-145, which regulates the miR-145/TGF-β/Wnt signaling cascade.

ApoVs are generated specifically during cell apoptosis. These vesicles can encapsulate apoptosis releasing cellular factors and can be engulfed by various cell types, including fibroblasts, macrophages, and specific phagocytes such as Sertoli cells, for the purpose of clearance [56, 57]. Furthermore, the phagocytosis of apoptotic cells may promote macrophages to render molecular memory [58]. Although the importance of ApoVs in regulating tissue homeostasis is widely recognized, the specific mechanisms by which they rescue the function of diseased stem cells and regulate bone tissue homeostasis in diseases related to bone metabolism remain poorly understood. In this study, we pre-treated OVX MSCs with ApoVs derived from WT MSCs in vitro. The systematically injected ApoV-preconditioned OVX MSCs achieved similar reparative effects as WT MSCs, opening up the possibility of reusing these cells for stem cell therapy. This method does not involve direct gene-level intervention, thereby eliminating a range of ethical and biosafety concerns and presenting promising prospects for application. Specifically, the OVX MSCs, pre-conditioned with ApoVs derived from WT MSCs, obviously elevated the bone density and trabecular bone volume in the distal femurs. They also inhibited the differentiation of CD3^+^T cells and Th1/Th17 cell subsets, reduced the expression of related inflammatory factors, and promoted the differentiation of Treg cells, thereby reversing the state of immune hyperactivation observed in OVX-induced osteoporotic mice. These results align with the theory that immune cell alterations contribute to the pathogenesis of postmenopausal osteoporosis in addition to the direct negative impact of estrogen deficiency on bone homeostasis [59]. Additionally, the ApoVs inhibited adipogenic differentiation and promoted osteogenic differentiation of MSCs derived from OVX mice, restoring their differentiation capacity to a relatively normal level. However, the OVX MSCs alone did not exhibit a similar therapeutic effect. The maintenance of physiological functions in MSCs relies on the homeostasis of their microenvironment [60]. We hypothesize that the impairment of OVX MSCs may be attributed to long-term exposure to high levels of proinflammatory factors in vivo, such as TNF-α and IFN-γ [61-63]. Additionally, a prolonged deficiency of estrogen can disrupt the balance between MSC differentiation into osteoblasts and adipocytes. Furthermore, the function of MSCs can be affected by the excessive use of anti-osteoporosis drugs in clinical patients, thereby increasing the risk of secondary osteoporosis [64]. If stem cells from patients prove ineffective in treating their own diseases, the reliance on MSCs derived from healthy individuals is inevitable. However, this situation presents challenges due to limited cell sources and the risk of immune rejection, which hinder the application and development of stem cell therapy technology. Conversely, if we can devise an approach to repair damaged stem cells in vitro and restore their therapeutic function, it will greatly advance the progress of stem cell therapy and bring hope to the majority of patients. Moreover, the significant ability of apoVs to restore multidirectional differentiation capacity in diseased stem cells suggests promising opportunities for their application in tissue engineering and regenerative medicine. Nonetheless, further research is needed to explore these possibilities.

While ApoVs derived from WT MSCs successfully restored the impaired therapeutic capability, immunoregulatory property, and differentiation potential of OVX MSCs, the specific mechanism underlying this effect has yet to be fully understood. Multiple hypotheses exist regarding the damage of MSCs in OVX mice. Some researchers propose that estrogen deficiency-induced T-cell hyperactivation promotes osteoclast differentiation, leading to reduced bone mineral density and increased bone resorption [65-68]. While our previous study proved that high levels of IFN-γ and TNF-α in OVX mice cooperatively activated the NF-κB/Smad 7 signaling pathway in MSCs, resulting in functional defects and long-term promotion of MSC tumorigenesis through NF-κB-mediated oncogene activation [61]. Our findings indicate that increased expression of TGF-βR2 mediates the activation of the TGF-β/Smad 2/3 signaling pathway, which subsequently upregulates DKK-1, an inhibitory protein of the Wnt/β-catenin signaling pathway. This cascade ultimately suppresses the expression of active β-catenin, inhibiting the Wnt/β-catenin pathway and negatively regulating the osteogenic differentiation of OVX MSCs. Surprisingly, WT apoVs downregulate the expression of TGF-βR2, leading to the inhibition of the TGF-β/Smad 2/3 pathway, upregulation of DKK-1 expression, promotion of activated-state β-catenin expression, activation of the Wnt/β-catenin pathway, and restoration of impaired osteogenic differentiation and immunoregulatory capacity in OVX MSCs. The TGF-β/Smad 2/3 and Wnt/β-catenin pathways are recognized as critical signaling pathways in regulating osteogenesis and contributing to the maintenance of bone homeostasis [69, 70]. The dysregulation of these two pathways has implications for the treatment of various bone-related diseases and the management of clinical symptoms [71-74]. Recent research has shown that the activation status of these pathways can be influenced by miRNA in the OVX model, with varying degrees of regulation in different locations. For example, the TGF-β pathway is primarily regulated in the jaw, whereas the Wnt pathway is mainly regulated in the femurs [75]. Kuniaki et al. demonstrated that TGF-β1 promotes the expression of Wnt10 in osteoclasts through the phosphorylation of Smad 2/3, leading to the activation of the Wnt pathway and enhancing the supportive effect on the osteoblast mineralization process [76]. Furthermore, Guerrero et al. found that TGF-β inhibited the osteoblast-like differentiation of rat vascular endothelial cells caused by high phosphate levels by suppressing the BMP pathway and the Wnt/β-catenin pathway. Stimulation of human MSCs with TGF-β results in the rapid co-translocation of Smad 3 and β-catenin into the intracellular nucleus, jointly modulating the expression of various genes. Overexpression of Axin, an inhibitory protein for β-catenin, activates the TGF-β pathway by presenting Smad 3 to the TGF-β receptor 1 [77]. These studies suggest that the interaction between the TGF-β and Wnt pathways plays a crucial role in mediating various physiopathological activities. Our data demonstrated that the hyperactivation and inhibition of the TGF-β pathway were responsible for impairing the osteoblastic differentiation capacity of OVX MSCs and for the restorative effect of apoVs. We proposed that this impairment may be related to the abnormal activation of the TGF-β/Smad 2/3 pathway under chronic inflammatory conditions [78, 79]. Additionally, previous reports have indicated that elevated TGF-β1 in patients with chronic inflammation can affect osteogenesis by activating the TGF-β/Smad 2/3 pathway [80, 81], suggesting that the chronic inflammatory state observed in osteoporosis may also promote TGF-βR2 expression through certain mechanisms, thereby activating the TGF-β pathway and mediating injury to MSCs. However, further study is required to explore these mechanisms.

In our previous report, we observed that overexpression of IFN-γ and TNF-α in OVX mice led to elevated expression of Smad 7, a repressor protein of the TGF-β pathway [61]. However, in this study, we found that the TGF-β/Smad 2/3 pathway was activated in OVX MSCs. These seemingly contradictory results can be explained from two perspectives. Firstly, Smad 7 inhibits the TGF-β pathway by binding to TGF-β receptor 1, thereby preventing recruitment and phosphorylation of Smad 2/3. Additionally, TGF-β receptor 1 requires binding with receptor 2 to initiate its function. In our study, we did not observe significant changes in TGF-β receptor 1 expression levels, while increased TGF-β receptor 2 expression mediated the activation of the TGF-β pathway and the inhibitory effect on the downstream Wnt pathway, acting through a distinct mechanism. Secondly, hyperactivation of the TGF-β pathway in OVX MSCs may trigger feedback regulation, leading to a transient increase in Smad 7 expression, which serves as a protective mechanism for the body. However, the specific mechanisms underlying the regulation of TGF-β receptor by WT apoVs treatment remain unclear and will be investigated in future experiments.

miRNAs play diverse roles during different stages of bone formation and are implicated in the pathological processes of various bone metabolism-related diseases [82-87]. The role of miR-145 in bone metabolism is currently a subject of controversy. Some researchers argue that miR-145 promotes osteogenesis and suppresses osteoclastogenesis, thereby participating in both physiological and pathological bone metabolism activities, including osteoporosis [88]. On the other hand, there are scholars who support the notion that miR-145 has a negative impact on osteogenesis and may contribute to the development of osteoporosis [89]. These contrasting findings suggest that miR-145 exhibits different effects on bone homeostasis under different physiological and pathological conditions. This variability could be attributed to the diversity of cell types involved and the local microenvironment. In our study, we discovered that the downregulation of miR-145 in OVX MSCs upregulated TGF-βR2, subsequently activating the TGF-β/Smad 2/3 pathway and initiating downstream cascade reactions that regulate the biological function of MSCs. This finding may be linked to the osteopenic phenotype observed in OVX mice. Another investigation reported that in systemic sclerosis models, miR-145 directly inhibits the expression of Smad 2/3 mRNA, thereby inhibiting the TGF-β/Smad 2/3 pathway [90]. These findings suggest the involvement of multiple mechanisms in miR-145-mediated regulation of the TGF-β/Smad 2/3 pathway.

In this study, we observed that WT apoVs efficiently delivered miR-145 directly to OVX MSCs. This, in turn, led to the restoration of these cells to a relatively healthy state, thereby enhancing their therapeutic capacity for osteoporosis. However, the insufficient levels of miR-145 in OVX apoVs significantly limited their ability to restore OVX MSCs. This limitation may be attributed to the fact that EVs, including apoVs, inherit most functional and bioactive substances from their parental cells, allowing them to maintain their signaling and regulatory abilities [91]. In the case of OVX MSCs, the lack of miR-145 prevents the packaging and formation of normal apoVs containing adequate levels of miR-145 during the apoptosis process. However, further investigation is required to understand how the microenvironment of osteoporosis influences the expression of miR-145 in MSCs.

**Figure 8:**
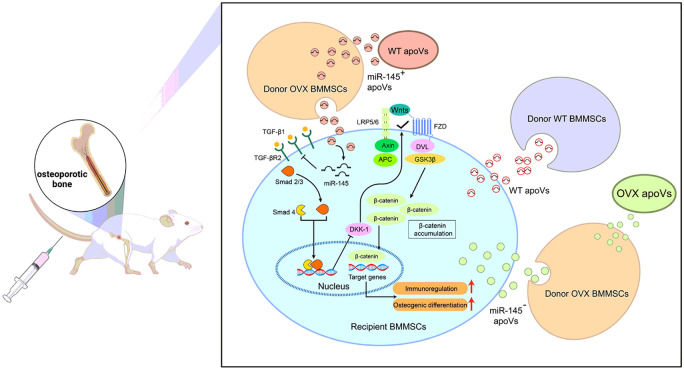
A schematic diagram for the mechanism underlying the apoVs-mediated rescue of the impaired OVX MSCs biological function and their capacity to improve osteoporosis. Systemic infusion of WT MSCs or WT apoVs preconditioned OVX MSCs rescue the impaired recipient MSCs via the direct cargo and reuse of miR-145a-5p initiating modulation of TGF-β1/Smad 2/3 and Wnt/β-catenin signaling pathway, thus providing a promising novel approach of exploring apoVs-based MSCs engineering and expanding the application scope of stem cell therapy for osteoporosis.

In summary, Fig. 8 portrays the schematic representation of the therapeutic effect of apoVs mediated by the miR-145 delivery. The reduced expression of miR-145a-5p in OVX MSCs adversely affected osteogenic differentiation and immunoregulation capacity through its impact on the TGF-β/Smad 2/3-Wnt/β-catenin axis. Notably, WT MSCs-derived apoVs successfully restored these biological functions by delivering the cargo and reutilizing the miR-145a-5p in OVX MSCs. This phenomenon greatly facilitates the application of apoVs in osteoporosis therapy. However, there are still several unanswered questions that require further investigation. Firstly, it is crucial to explore the specific changes in apoVs derived from osteoporotic MSCs and understand the underlying mechanisms. Additionally, it is important to determine if there are other key molecules involved in the rescue of osteoporotic MSCs by apoVs. Therefore, a comprehensive profiling of osteoporotic MSCs before and after apoV treatment would be beneficial for the targeted optimization of MSC modification in the future.

## Conclusions

In this study, we explored the molecular mechanism of MSCs damage in osteoporosis and proposed a new approach utilizing apoVs to rescue the impaired MSCs and their therapeutic capacity for osteoporosis by restoring miR-145a-5p deficiency and dissected the underlying mechanisms. Our findings highlighted the significance of the miR-145-TGF-β/Smad 2/3-Wnt/β-catenin axis in this process, suggesting a novel mechanism for osteoporotic stem cell damage and presenting a non-genetic engineering approach to modifying stem cells, particularly those derived from patients. Consequently, our findings expanded the potential cell sources and enhanced the technology of stem cell therapy by making it feasible to utilize damaged stem cells from patients.

## Materials and Methods

### Mice

Female C57BL/6 mice (JAX #000664), obtained from the Jackson Laboratory, were used in this study. All procedures involving animal subjects were conducted in strict accordance with the approved guidelines and under the protocols sanctioned by the Institutional Animal Care and Use Committee (IACUC) of the University of Pennsylvania (#805478).

### Isolation of mouse BMMSCs

BMMSCs were isolated from the limbs of C57BL/6 mice, and adherent cells were cultured for two passages to enrich bone marrow mesenchymal stem cells (BMMSCs) according to the method previously described [22, 92]. Briefly, a suspension containing 1.5×10^7^ nuclear cells from bone marrow was prepared and seeded onto 10 cm culture dishes. These dishes were placed in an incubator at 37°C with a 5% CO_2_ atmosphere. After 48 h, non-adherent cells were removed, leaving the adherent cells, which were subsequently cultured for an additional 16 days. The culture medium used was alpha Minimum Essential Medium (α-MEM) supplied by Invitrogen, USA, enhanced with 20% fetal bovine serum (FBS, Gemini Bio, USA), 2 mM L-glutamine, 55 μM 2-mercaptoethanol, and a combination of 100 U/ml penicillin and 100 μg/ml streptomycin, all sourced from Invitrogen (USA). To minimize hematopoietic cell contamination, the adherent cell colonies were passaged once, with frequent medium changes. For experimental purposes, MSCs from the third to fifth passages were utilized.

### Induction of MSCs apoptosis and isolation of apoVs

MSCs were induced to undergo apoptosis using a modified protocol based on our previous report [93]. Upon reaching full confluency, the MSCs were treated with a basic medium devoid of FBS, but containing 500 nM staurosporine (STS, Enzo Life Sciences, USA) for 16 h. Post-treatment, the cells were examined under a microscope. For the assessment of morphological changes and the expression of apoptosis-related markers, the cells underwent immunofluorescent co-staining utilizing cleaved caspase-3 (Cell Signaling Technology, USA) and Hoechst 33342 (Sigma-Aldrich, USA).

Subsequently, apoVs were isolated using an optimized gradient centrifugation method, detailed in Fig. S2. The procedure commenced with sequential centrifugation: initially at 800 g for 10 min, followed by 2000 g for another 10 min, both conducted at 4°C. These steps were designed to remove apoptotic cell debris. Subsequently, the supernatant, which contained EVs, was collected. To isolate apoVs, this supernatant was further centrifuged at 16,000 g for 30 min at 4°C. The resulting apoVs were then washed once with PBS and resuspended in either PBS or fresh culture medium for subsequent experimental applications.

### Characterization of MSCs derived apoVs

Transmission electron microscopy (TEM) was employed to observe the morphology of apoVs. ApoV pellets, obtained post-centrifugation, were fixed in 1% glutaraldehyde (Sigma-Aldrich, USA) for 30 min at 4°C. Then, 10 μl of the apoV sample was placed on carbon-coated formvar copper grids (ProSciTech, Australia) at room temperature. After a minute, the samples were stained with 2.5% uranyl acetate (Electron Microscopy Sciences, USA) for 3 min. Images of the apoVs were captured using a JEM-1200EX electron microscope (JEOL, Japan).

For nanoparticle tracking analysis (NTA), the apoVs were initially diluted in PBS and further diluted with ultrapure water at a ratio of 1:1000. The concentration, particle size, and zeta potential of the apoVs were measured utilizing a ZetaView PMX120 (Particle Metrix, Germany). The obtained data were analyzed with the ZetaView software (version 8.02.31).

For immunofluorescent staining of apoVs, the vesicles were initially fixed in 2% paraformaldehyde (PFA) for 30 min and blocked utilizing 5% BSA for 1 h. The apoVs were then incubated overnight at 4°C with primary antibodies targeting integrin alpha-5, calreticulin, calnexin, and Annexin V (all sourced from Cell Signaling Technology, USA). Post-incubation, the apoVs were washed with PBS and centrifuged at 16,000g for 30 min. The apoV pellets were subsequently incubated for 1 h with an Alexa Fluor 488-conjugated secondary antibody (Invitrogen, USA). After a final wash and centrifugation, the apoVs were counterstained for cytomembranes with CellMask. Imaging was performed using a Zeiss Elyra 7 with Lattice SIM.

For nano flow cytometry (nFCM) detection, freshly isolated apoVs suspended in PBS were incubated with antibodies against integrin alpha-5, calreticulin, and calnexin. Following a previously established protocol, this step was done to analyze the surface molecules [94]. The standard curve and acquisition parameters, including detector gain, laser power, and detection threshold, were set up. The apoVs were then introduced into the nFCM instrument (NanoFCM, Xiamen, China). Analysis of the total number and the proportion of positively stained particles was conducted using the NanoFCM software (NanoFCM Professional Suite V1.15).

### In vitro ApoVs stimulation and systemic infusion of MSCs

Cells were plated at a density of 6×10^5^ cells per well in a 6-well plate. Once the cells were adherent, the medium was replaced with complete α-MEM supplemented with apoptotic vesicles (apoVs), maintaining a cell-to-apoV ratio of 1:50. The cells were then incubated at 37°C in an atmosphere containing 5% CO_2_ for 72 h. Following this incubation, MSCs, both with and without *in vitro* stimulation by apoVs, were harvested, resuspended in PBS at a concentration of 4×10^6^ cells per 200 μL, and administered intravenously via the tail vein on a weekly basis. After a four-week treatment period, the mice were euthanized for further analysis.

### X-ray scanning

The femurs were excised from mice and then fixed in 4% PFA. The fixed bones were immersed in PBS for a day to remove residual PFA. Following this, the femurs underwent a dehydration process using a graded series of ethanol. Any excess tendon and cartilage tissues were carefully trimmed using ophthalmic scissors. This trimming was essential to ensure stable positioning of the femurs in the X-ray scanner (Carestream Health, Canada) for subsequent radiographic scanning and imaging.

### Micro CT analysis

The preparation of femur specimens followed the previously mentioned pretreatment procedures. The femurs were imaged and analyzed using a high-resolution Scanco μCT35 scanner (Scanco Medical AG, Switzerland), as detailed in our prior study [22]. Briefly, the specimens focusing on the metaphysis of femurs were scanned utilizing a voxel size of 20 μm at 200 μA and 70 kVp. A region of interest, specifically targeting the trabecular bone, was manually outlined near the endocortical surface. The scanned data were then reconstructed using Scanco software. For image reconstruction, datasets were imported into Amira 5.3.1 software (Visage Imaging, Germany). Parameters such as the bone volume to total volume ratio (BV/TV) and bone mineral density (BMD) were also computed.

### Hematoxylin and eosin (H&E) staining

Femurs from each experimental group were harvested and initially fixed in 4% PFA. Following fixation, the bones were decalcified using 5% EDTA. The decalcified femurs were then embedded in paraffin and sectioned into slices with a thickness of 6 μm. These sections were stained using hematoxylin and eosin (H&E) for histological examination. The stained sections were analyzed with a focus on trabecular bone using ImageJ software (National Institutes of Health, USA).

### Enzyme-linked Immunosorbent (ELISA) Assay

Peripheral blood serum was collected from mice and analyzed for the expression levels of TNF-α, IFN-γ, and IL-17 proteins using the Mouse ELISA Ready-SET-GO kits (eBioscience, USA). Similarly, the supernatant from the MSC culture medium was also collected and assessed for TGF-β1 expression using the same Mouse ELISA Ready-SET-GO kit (eBioscience, USA).

### Culture of mouse splenocytes and T lymphocytes

Splenocytes were isolated from mouse spleens by finely grinding the tissue until no discernible splenic material remained and then straining the resultant suspension through a 70 nm cell strainer. Subsequently, ACK lysis buffer (Lonza Bioscience, Switzerland) was added for 5 min at room temperature to lyse red blood cells. This was followed by centrifugation to separate the splenocytes. Naive CD25^−^CD4^+^ T cells were then isolated from the splenocytes using magnetic cell sorting (MACS) technology (Miltenyi Biotec, Germany). For cell culture, the isolated T cells were maintained in RPMI 1640 medium (Lonza Bioscience, Switzerland) supplemented with 10% FBS (Sigma-Aldrich, USA), 10 mM HEPES (Lonza Bioscience, Switzerland), 1 mM sodium pyruvate (Gibco, USA), 55 μM 2-mercaptoethanol, Non-Essential Amino Acids (Gibco, USA), and 100 U/mL Penicillin and Streptomycin (Gibco, USA). T cell activation was achieved using plate-bound anti-mouse CD3 antibody (BioLegend, USA) and soluble anti-mouse CD28 antibody (BioLegend, USA), both at a concentration of 2 μg/ml.

### Th1/Th17 subsets and Treg cells differentiation

To induce differentiation into Th1/Th17 subsets and Treg cells, naive CD25^−^CD4^+^ T cells were stimulated with anti-CD3/CD28 antibodies in a polarizing environment, as described previously [95]. The specific conditions for each subset were as follows, with a duration of 3 days for the differentiation process:

#### Th1 Polarization:

The medium was supplemented with IL-12 (20 ng/ml, R&D Systems, USA) and anti-IL-4 neutralizing antibodies (10 μg/ml, BioLegend, USA).

#### Th17 Polarization:

The cells were cultured in the presence of IL-6 (50 ng/ml, R&D Systems, USA), TGF-β1 (2 ng/ml, R&D Systems), along with both anti-IFN-γ (10 μg/ml, BioLegend, USA) and anti-IL-4 neutralizing antibodies (10 μg/ml, BioLegend, USA).

#### Treg Polarization:

This required TGF-β1 (2 mg/mL; R&D Systems, USA), IL-2 (2 mg/mL, R&D Systems, USA), and the same concentrations of anti-IFN-γ and anti-IL-4 neutralizing antibodies as used for Th17 polarization.

### Flow cytometry

For the characterization of MSCs, the isolated cells were incubated with a panel of antibodies: CD34, CD45, CD73, CD90, CD105, CD126, CD166, and Sca-1 (all from BioLegend, USA). This incubation was carried out in the dark at 4°C for 30 min. Following incubation, the cells were washed with a washing solution to remove any residual antibodies. They were then resuspended and fixed in PBS containing 2% PFA. The samples were subsequently analyzed using a FACS Calibur Flow Cytometer (BD Biosciences, USA) to determine the percentages of positive expression for the aforementioned markers. Data analysis was performed using FlowJo v10 software (FlowJo LLC, USA).

To evaluate apoptosis in MSCs, including those stimulated with STS, the cells were first suspended in a binding buffer. They were then incubated with Annexin V (BD Pharmingen, USA) and 7-AAD (BioLegend, USA) for 15 min in the dark at room temperature. To halt the reaction, an equivalent volume of binding buffer was added. Apoptosis rates were then assessed using a FACS Calibur Flow Cytometer (BD Biosciences, USA). The gathered data were analyzed with FlowJo v10 software (FlowJo LLC, USA).

For the analysis of Th1/Th17 subsets and Treg cells, cells were harvested and first incubated with CD4 and CD25 antibodies (BioLegend, USA) for 45 min on ice in dark conditions. Subsequently, the cells were stained with specific antibodies: IFN-y for Th1, IL-17 for Th17, or Foxp3 for Treg cells (all from BioLegend, USA). For cell fixation and permeabilization, a Foxp3 Staining Buffer Kit (BioLegend, USA) was utilized. The stained cells were then analyzed using a FACS Calibur Flow Cytometer (BD Biosciences, USA). The acquired data were processed and analyzed with FlowJo v10 software (FlowJo LLC, USA).

### Cell proliferation assay

MSCs were plated on chamber slides at a density of 1×10^4^ cells per well and cultured for 3 days. Subsequently, the cells were incubated with a BrdU solution (1:100, Invitrogen, USA) for 20 h. After incubation, BrdU staining was performed using the BrdU Staining Kit (Invitrogen, USA). The proportion of BrdU-positive cells was calculated as a percentage of the total cell count based on eight different image fields per sample. This assay was replicated in five independent samples for each experimental group.

### Population doubling (PD) assay

Single colony cluster P0 cells, at their first passage, were seeded at a density of 5×10^5^ cells on a 60 mm dish (Corning, USA). Upon reaching confluence, the cells were harvested and reseeded at the same density. The PD number was calculated using the formula PD = log_2_ (number of harvested cells/number of seeded cells). This calculation was based on the cumulative total of cells obtained from each passage, continuing until the cells ceased dividing. To ensure reliability, this assay was conducted with five independently isolated cell samples for each experimental group.

### Osteogenic differentiation of MSCs

MSCs were cultured in an osteogenic inductive medium, which comprised 100 μM L-ascorbic acid 2-phosphate, 2 mM β-glycerophosphate, and 10 nM dexamethasone (all sourced from Sigma-Aldrich, USA). Ten days after induction, total proteins were extracted from the cells, and the expression of osteogenic-associated proteins was assessed via western blot analysis. Furthermore, at 21 days post-induction, the cells underwent staining with 1% Alizarin Red-S (Sigma-Aldrich, USA) to detect mineralized nodules. The extent of mineralization was quantified using Image J software (NIH, USA), with the results presented as the percentage of the total area covered by the positive staining.

### Adipogenic differentiation of MSCs

MSCs were cultured in an adipogenic inductive medium composed of 500 nM hydrocortisone, 60 μM indomethacin, 500 nM isobutylmethylxanthine, 10 μg/ml insulin, and 100 nM L-ascorbic acid phosphate, all obtained from Sigma-Aldrich, USA. Seven days following the induction, total proteins were extracted from the cells for the purpose of detecting adipogenic-associated proteins via western blot analysis. Additionally, 21 days post-induction, the cells were stained with Oil Red O (Sigma-Aldrich, USA) to visualize lipid droplets, indicative of adipogenic differentiation. The positively stained cells were observed under a microscope, and the results were quantified as the proportion of oil-red O-positive cells relative to the total cell count.

### Western blot analysis

Total protein was extracted from the cells using the M-PER Mammalian Protein Extraction Reagent (ThermoFisher, USA). The protein concentrations were then determined using a BCA Protein Assay Kit (ThermoFisher, USA). For the western blot analysis, 20 μg of the extracted proteins were separated on NuPAGE BT gels (Invitrogen, USA) and subsequently transferred onto Immobilon^™^-P PVDF membranes (Millipore, USA). The membranes were blocked with a solution containing 0.1% Tween 20 and 5% non-fat milk for 1 h. They were then incubated overnight at 4°C with primary antibodies at dilutions ranging from 1:200 to 1:1000. These antibodies targeted various proteins, including ALP, PPAR-γ, LPL, phospho-Smad2/3 (Santa Cruz, USA), Dickkopf 1 (Sigma-Aldrich, USA), active β-catenin, β-catenin (Millipore, USA), phospho-ERK, ERK (ThermoFisher, USA), Smad 2/3, phospho-mTOR, mTOR, p85, p110, phospho-p65, p65 (Cell Signaling Technology, USA), Runx2, β-actin, TGF-β receptor 1, and TGF-β receptor 2 (Abcam, USA). After primary antibody incubation, horseradish peroxidase-conjugated IgG (1:10,000 dilution, Santa Cruz, USA) was applied for 1 h. Detection was enhanced using a SuperSignal West Pico Chemiluminescent Substrate (ThermoFisher, USA). The bands were visualized on films (Bioland, USA) and analyzed with Image J software (NIH, USA). β-actin was used as a loading control to quantify the amount of protein loaded.

### Induction of T cell apoptosis by MSCs

T cells were activated as described in section 4.10. Subsequently, activated T cells (1×10^6^ cells per well) were cocultured with MSCs (0.2×10^6^ cells per well) in a 24-well plate using a T cell culture medium. After a 3-day coculture period, the cells in the supernatant were collected. The apoptosis of T cells was then evaluated by identifying cells positive for both Annexin V and 7-AAD using a FACS Calibur Flow Cytometer (BD Biosciences, USA).

### Real-time polymerase chain reaction (PCR)

Total RNA, inclusive of small RNA, was extracted from the cells using the miRNeasy Mini Kit (Qiagen, Germany). The quantification and purity assessment of the extracted total RNA were carried out using a NanoDrop ND2000 (ThermoFisher, USA). For cDNA synthesis, the miScript II RT Kit (Qiagen, Germany) was employed. The expression of miR-93–5p, miR-145a-5p, miR-294–3p, and pri-miR-145 was then quantified through real-time PCR, utilizing the miScript SYBR Green PCR Kit (Qiagen, Germany). This procedure included the use of the miScript Primer Assay and miScript Precursor Assay (Qiagen, Germany), which incorporated specific primers designed based on the latest sequences in miRBase. GAPDH and snRNA U6 were used as endogenous controls for pri-miRNA and mature miRNA expression in MSCs for normalization. The real-time PCR detection was conducted on a CFX96^™^ Real-Time PCR System (Bio-Rad), and gene expression was calculated using the efficiency-corrected ΔΔCt method.

### MicroRNA mimics and inhibitors transfection

miR-145 mimics, inhibitors, and corresponding negative controls, provided by Genecopoeia, USA, were transfected into MSCs according to the manufacturer's recommended protocols.

### Statistics

Data are presented as means ± standard deviation (SD), based on a minimum of three independent measurements, unless specified otherwise. Statistical analyses were performed using GraphPad Prism 9 software (GraphPad, USA). For comparisons between the two groups, the independent Student's t-test was employed. For multiple group comparisons, one-way analysis of variance (ANOVA) with Tukey’s post hoc analysis was utilized. A p-value of less than 0.05 was considered statistically significant.

## Data Availability

All data generated or analyzed during this study are included in this manuscript.
